# Effects of ischemic preconditioning on lower-limb anaerobic performance and neuromuscular adaptations

**DOI:** 10.7717/peerj.21296

**Published:** 2026-06-08

**Authors:** Ruibo Chen, Danyang Li, Binbin Jia, Yueqiang Ma

**Affiliations:** 1 Wuhan Sports University, Wuhan, Hubei, China; 2 Capital University of Physical Education and Sports, Beijing, China

**Keywords:** Ischemic preconditioning, Anaerobic capacity, Heart rate, Surface electromyography, Functional near-infrared spectroscopy

## Abstract

**Background:**

This study examined whether ischemic preconditioning (IPC) improves lower-limb anaerobic performance and neuromuscular function in female athletes.

**Methods:**

Twenty-two female second-level athletes were randomly assigned to an IPC or sham group in a single-blind, parallel design. Participants received once-daily IPC (220 mmHg, 4 × 5-min ischemia/5-min reperfusion) or sham intervention for seven days. Before and after the intervention, we measured 30-s Wingate performance, heart rate, blood lactate, surface electromyography of the lower-limb muscles, and oxyhemoglobin (HbO) in the dorsolateral prefrontal cortex (DLPFC). A 2 (group: IPC *vs.* sham) × 2 (time: pre *vs.* post) repeated-measures analysis of variance was used.

**Results:**

Compared with sham, IPC induced greater increases in average power and total work (*P* < 0.05), whereas peak power and fatigue index did not differ significantly between groups (*P* > 0.05). Additionally, there were no between-group differences in mean or peak heart rate , blood lactate, the median frequency of the lower-limb muscles, or DLPFC HbO changes (*P* > 0.05).

**Conclusion:**

Seven days of IPC induced a modest enhancement in lower-limb anaerobic performance in female athletes without substantially altering electromyography, heart rate, blood lactate responses, or cortical oxygenation levels. IPC may be a practical, safe, and effective non-pharmacological strategy to support training preparation in competitive athletes.

## Introduction

With the increasing scientification of high-performance training, the marginal gains achievable through traditional training methods alone have gradually diminished. Consequently, identifying novel adjunctive strategies to optimize athletic performance has become a major focus in contemporary strength and conditioning practice. In recent years, utilizing limb blood flow modulation techniques to enhance athletic performance has shown tremendous potential, particularly blood flow restriction training (BFRT) and ischemic preconditioning (IPC) (Patterson, Burr & Warmington, 2021). As an alternative to high-intensity resistance training, BFRT has been proven by numerous studies to improve athletes’ maximal muscle strength and explosive power (Wang et al., 2023; Li et al., 2025). In contrast to BFRT, which applies blood flow restriction during exercise, IPC is primarily administered prior to exercise and offers significant advantages as an ergogenic aid in improving both aerobic and anaerobic capacities (Incognito, Burr & Millar, 2016). Ischemic preconditioning (IPC) is a technique designed to enhance exercise performance by applying a tourniquet to the proximal portion of a limb before exercise, thereby inducing alternating brief periods of blood flow occlusion and reperfusion (Marocolo et al., 2017). The potential mechanisms underlying the ergogenic effects of IPC are primarily attributed to humoral processes, which involve the local release of substances into the bloodstream during reperfusion, and neural pathways, where metabolites synthesized during transient ischemia locally activate afferent nerves (Souza et al., 2025). Existing studies have reported that IPC can enhance sport-specific performance in several disciplines, including cycling (Kilding, Sequeira & Wood, 2018), sprinting (Chen et al., 2023), and swimming (Marocolo et al., 2018).

Anaerobic capacity is a key determinant of athletic performance, not only in short-duration, high-intensity events but also in the final sprint phase of endurance competitions (Noordhof, Skiba & De Koning, 2013). It reflects the ability of the phosphagen and glycolytic systems to support external work. It is constrained by factors such as the efficiency of metabolic by-product clearance and the pattern of neuromuscular recruitment (Plisk, 1991). Accordingly, improving anaerobic capacity requires both optimising the intramuscular metabolic environment and enhancing neuromuscular function. Previous work suggests that IPC may favourably modulate cellular energy metabolism by activating protein kinase C signalling and opening mitochondrial Adenosine Triphosphate (ATP)-sensitive potassium channels, thereby promoting ATP synthesis and attenuating its breakdown (Takeo & Nasa, 1999). However, whether IPC can reliably enhance anaerobic performance remains unclear, and robust evidence from controlled trials is still lacking. On the one hand, many existing studies have employed a single, acute bout of IPC and failed to demonstrate meaningful improvements in short-term high-intensity performance (Lalonde & Curnier, 2015; Chen et al., 2024). This raises the possibility that the ergogenic benefits of IPC may depend on a dose–response relationship, with repeated IPC stimuli being necessary to induce more persistent skeletal muscle adaptations. On the other hand, most prior research has focused primarily on external performance outcomes, with limited assessment of the underlying neuromuscular adaptations induced by IPC.

Accordingly, the current study aimed to assess the impact of seven days of IPC on lower-limb anaerobic performance, neuromuscular adaptations, and prefrontal cortical oxygenation in female athletes. Based on the existing literature, we hypothesized that, compared with the sham IPC condition, the seven-day IPC intervention would induce the following effects: (1) A significant improvement in lower-limb anaerobic performance during the Wingate Anaerobic Test; (2) Enhanced lower-limb muscular endurance during high-intensity exercise; (3) Higher heart rate responses and lower blood lactate concentrations during exercise; (4) Increased cortical oxyhemoglobin (HbO) concentration during exercise.

## Materials & Methods

### Participants

Twenty-two female Sanda athletes with a national second-level performance certification were recruited for this study. We specifically recruited female Sanda athletes for this study because competitive Sanda involves repeated high-intensity lower limb kicking and striking, which predominantly rely on anaerobic metabolism for energy supply (Zhao et al., 2006). The required sample size was estimated using G*Power 3.1.9.7. Assuming a statistical power of 0.80, an alpha level of 0.05, and an effect size of 0.35 (Paradis-Deschênes et al., 2020), a minimum of 20 participants was required. To account for an anticipated 10% dropout rate, 22 participants were ultimately enrolled. Participants underwent three routine training sessions per week, each lasting 120 min and consisting of moderate-to-high-intensity sport-specific technical training. Inclusion criteria were as follows: (1) age between 18 and 25 years; (2) possession of a national second-level or higher athlete certificate; (3) at least 3 years of systematic Sanda training experience; and (4) voluntary participation with signed informed consent. Exclusion criteria were: (1) sports injuries within the past 6 months, such as ligament tears, fractures, or joint dislocations; (2) cardiovascular, neurological, or other medical conditions contraindicating IPC; (3) use of medications or dietary supplements within the past 3 months; and (4) inability to complete the full experimental protocol. To minimise potential confounding factors, participants were instructed to avoid strenuous exercise, alcohol, and caffeine for 24 h before each test session and to maintain adequate sleep on the night preceding testing. The study protocol was approved by the Medical Ethics Committee of Wuhan Sports University (2025113). All participants provided written informed consent and were informed of the procedures and safety precautions before participation.

## Experimental procedure

**Figure 1:**
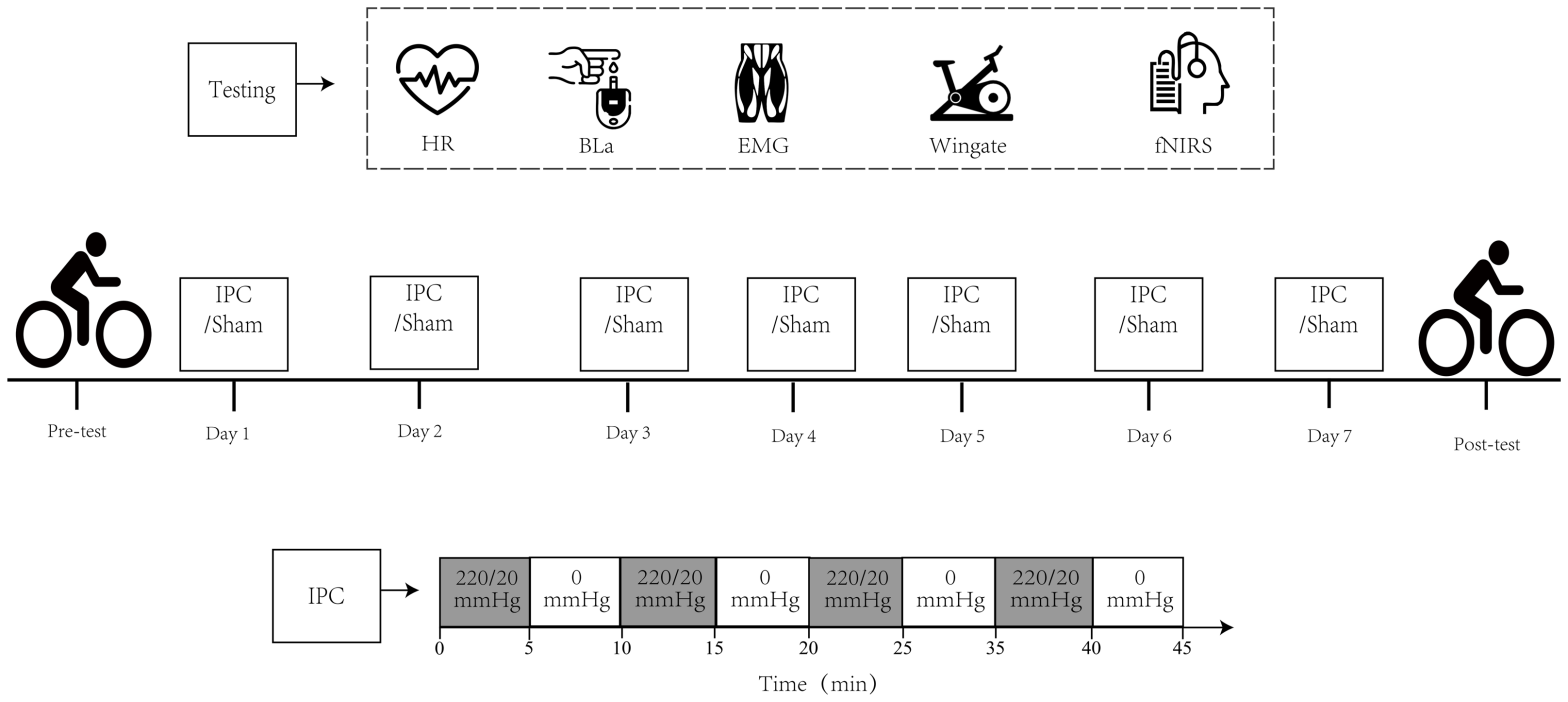
Experimental procedure. IPC, ischemic preconditioning; HR, heart rate; BLa, blood lactate; EMG, surface electromyography; Wingate, 30-s Wingate anaerobic test; fNIRS, functional near-infrared spectroscopy.

A randomised, single-blind, parallel-group design was used. Randomization was performed using the website http://www.randomizer.org, allocating participants to either the ischemic preconditioning (IPC) or sham intervention group. Before the formal experiment, all participants completed baseline assessments, including anthropometric measurements, and received standardised familiarisation with the Wingate testing procedures on a cycle ergometer. During the formal experiment, each participant underwent once-daily IPC or sham intervention for seven consecutive days. Throughout this intervention period, all participants maintained their routine training, with daily training volume and intensity strictly kept identical between the two groups under the coaches’ supervision. A 30-s Wingate anaerobic test was performed before and after the entire intervention period. During both pre- and post-intervention Wingate tests, external load variables (power output and total work) were recorded, along with internal load indicators, including heart rate, blood lactate (BLa), surface electromyography (EMG), and cerebral HbO concentration. The interval between the last IPC/sham session and the post-intervention testing session was 24 h. The overall experimental procedure is illustrated in Fig. 1.

### IPC intervention protocol

During the seven-day intervention period, participants received their assigned intervention protocol once daily at a fixed time, with a 24-hour interval between the final intervention session and the post-test. Specifically, the IPC protocol consisted of four cycles of 5-minute ischemia at 220 mmHg, each followed by 5 min of reperfusion (with no cuff pressure). The sham group followed the same schedule, but cuff pressure was set at 20 mmHg. Thus, the total duration of each IPC or sham session was 45 min (Marocolo et al., 2016). During the intervention, participants remained seated while two 10-cm-wide pneumatic cuffs (Theratools, China) were placed around the proximal portion of both thighs and inflated symmetrically. The IPC procedure is depicted in Fig. 1.

### Wingate anaerobic power test

Lower-limb anaerobic performance was assessed using a cycle ergometer (Monark 834E, Sweden) with the resistance load set at 0.075 kg kg^−1^ body mass (Castañeda-Babarro, 2021). Before each Wingate test, participants completed a 5-minute warm-up consisting of light pedalling interspersed with three 5-second all-out sprints, followed by 5 min of passive rest.

For the Wingate test, participants were instructed to pedal as fast as possible. Once the pedalling cadence reached 70 rpm, the preset resistance was automatically applied, and the 30-second test period began. Verbal encouragement was provided throughout the test. The following variables were recorded: peak power, average power, fatigue index, and total work.

### Heart rate and blood lactate measurements

Heart rate was monitored continuously during the Wingate test using a chest strap heart rate sensor (H10, Polar, Finland). The maximum heart rate (HRmax) and average heart rate (HRavg) during the 30-s test were extracted for analysis.

Capillary blood samples (0.2 μL) were obtained from the fingertip at rest and immediately after completion of the Wingate test. The second drop of blood was used in each sampling. Blood lactate concentration was measured using a portable lactate analyser (Lactate Scout, Germany), and the change in blood lactate (ΔBLa) was calculated as the difference between post- and pre-test values.

### Surface EMG recording of lower-limb muscles

A wireless surface EMG system (Wave Plus, Cometa, Italy) was used to record muscle activity from eight muscles of the right lower limb during the Wingate test. The sampling frequency was set at 2,000 Hz, and the primary EMG outcome was the median frequency (MF) of the power spectrum. The monitored muscles included: vastus medialis (VM), rectus femoris (RF), vastus lateralis (VL), biceps femoris (BF), semitendinosus (ST), medial gastrocnemius (MG), lateral gastrocnemius (LG), and tibialis anterior (TA). Before electrode placement, the skin over each target muscle was cleaned with alcohol wipes and allowed to dry. EMG sensors were then affixed over the muscle belly using adhesive tapes and secured with elastic bandages. Electrode placement followed the Surface ElectroMyoGraphy for the Non-Invasive Assessment of Muscles recommendations to align the electrodes with the muscle fibre orientation at rest, thereby ensuring the acquisition of a high-quality EMG signal.

### fNIRS monitoring of prefrontal cortical oxygenation

**Figure 2:**
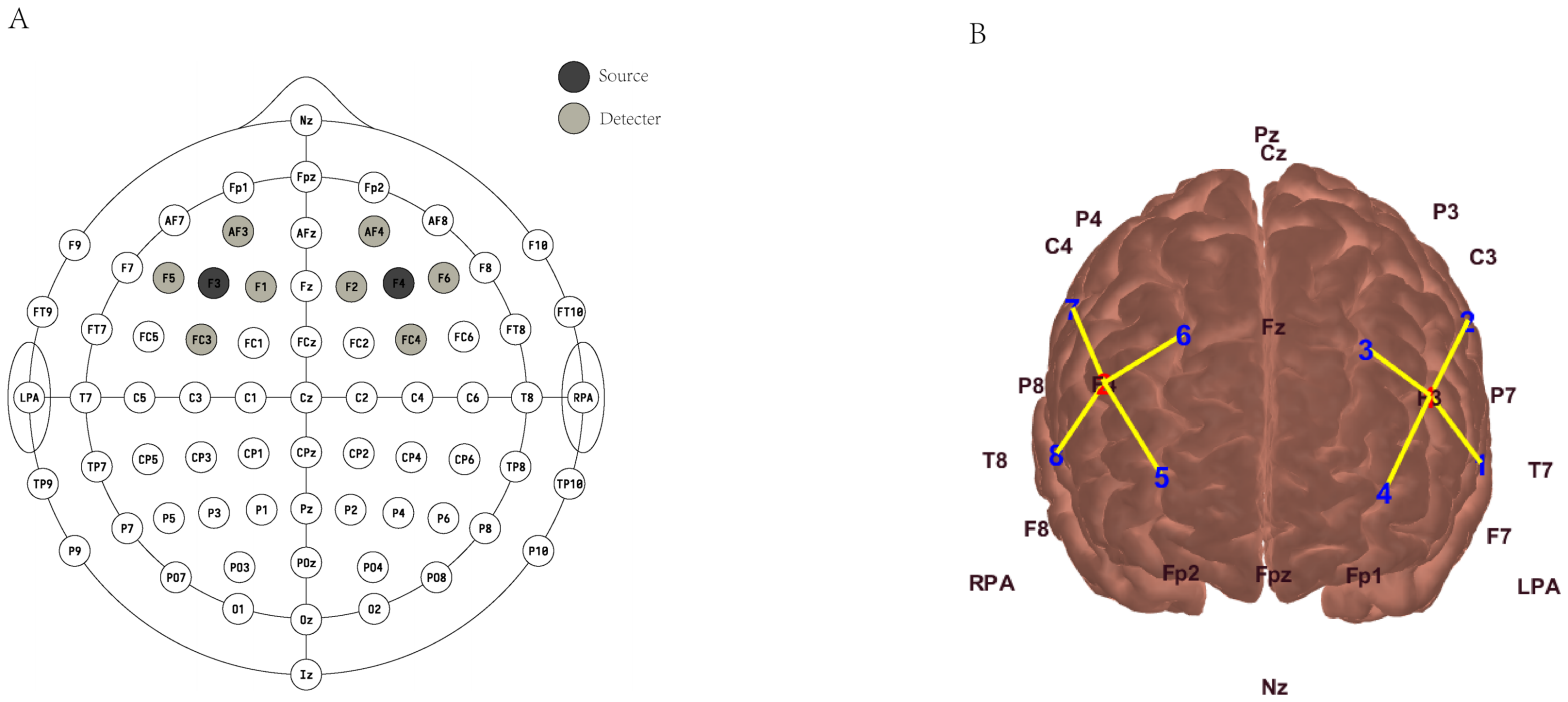
Optode montage of functional near-infrared spectroscopy (fNIRS) sources, detectors, and channels in the experiment.

A portable multichannel fNIRS system (NIRx-Sport2, NIRx, USA) was used to monitor changes in HbO concentration in the prefrontal cortex during the Wingate test. The system operated at wavelengths of 760–850 nm with a sampling frequency of 41 Hz. Two light sources and eight detectors formed eight channels covering the left and right dorsolateral prefrontal cortex (DLPFC), with a source–detector distance of 3 cm. Optode placement was determined using the fNIRS Optodes’ Location Decider tool, and positions were adjusted according to the international 10–10 EEG system with Cz as a reference point. For regional analysis, channels S1–D1, S1–D2, S1–D3, and S1–D4 were defined as the left DLPFC, and channels S2–D5, S2–D6, S2–D7, and S2–D8 as the right DLPFC. HbO data from each hemisphere were averaged to yield the mean left and right DLPFC signals. The optode and channel layout are shown in Fig. 2.

### Data processing

Surface EMG data were processed using MATLAB (MathWorks, Natick, MA, USA). The processing pipeline included DC offset removal, band-pass filtering (10–500 Hz, 4th-order Butterworth filter), full-wave rectification, and low-pass filtering at 6 Hz to obtain the linear envelope. Power spectral density was computed using the pwelch function, and the MF was extracted for analysis.

fNIRS data were preprocessed using the Homer3 toolbox (MATLAB). Channels with a signal-to-noise ratio <2 were excluded from further analysis (Kussman et al., 2016). Raw light intensity signals were converted to optical density, and motion artefacts were automatically detected when signal changes exceeded five times the mean or 50 times the standard deviation within a 0.5-s window. Motion artefacts were corrected using a wavelet-based algorithm with a threshold of 1.5 times the interquartile range. Optical density signals were then band-pass filtered (0.01–0.04 Hz) to remove low-frequency drift and high-frequency noise. Concentration changes in HbO were computed using the modified Beer–Lambert law. Finally, block averaging was performed within a −2 to 30 s time window relative to test onset to obtain mean HbO responses for statistical analysis.

### Statistical analysis

All statistical analyses were performed using SPSS 26.0 (IBM, Armonk, New York, USA). Data are presented as mean ± standard error (M ± SE). The Shapiro–Wilk test was used to assess normality, and Levene’s test was used to verify homogeneity of variance. A two-way repeated-measures analysis of variance (ANOVA) was conducted with group (IPC *vs.* sham) as the between-subjects factor and time (pre- *vs.* post-intervention) as the within-subjects factor to examine the effects of the seven-day intervention on peak power, average power, fatigue index, total work, HRavg, HRmax, ΔBLa, MF, and HbO. When a significant group × time interaction was detected, Bonferroni-corrected post hoc tests were performed. Effect sizes for ANOVA were expressed as partial eta squared (${\eta }_{\mathrm{p}}^{2}$), with values of ≥0.01, ≥0.06, and ≥0.14 interpreted as small, medium, and large effects, respectively (Cohen, 1988). The significance level was set at α = 0.05.

## Results

### Wingate anaerobic performance

**Figure 3:**
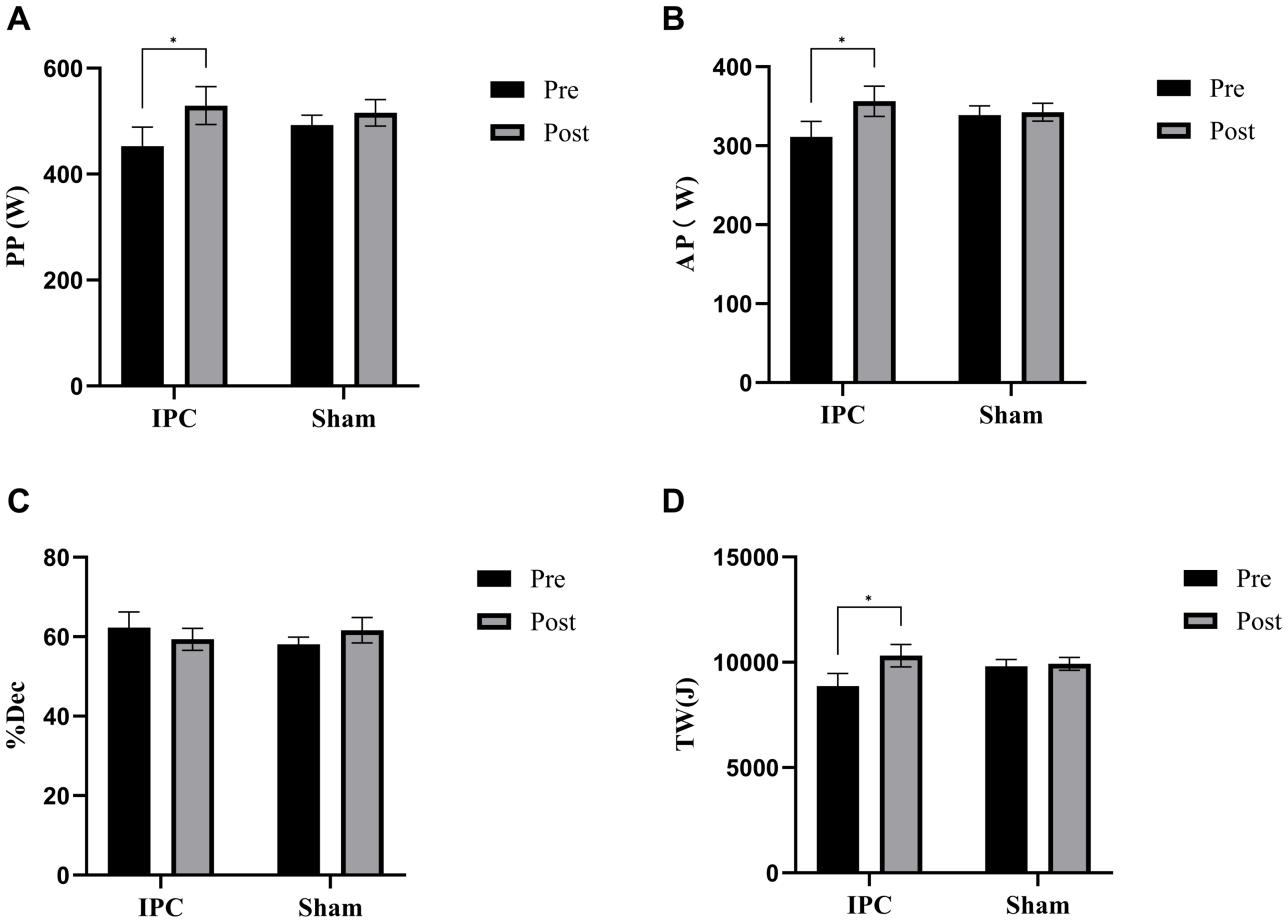
Results of lower-limb anaerobic performance in the Wingate test before and after IPC. (A) Peak power; (B) average power; (C) fatigue index; (D) total work. PP, peak power; AP, average power; %Dec, fatigue index; TW, total work. Data are mean ± SE. *Significantly different from pre-intervention (*p* < 0.05); the same notation applies to subsequent figures.

All 22 participants completed the full experimental protocol without dropouts. Figure 3 presents the changes in Wingate test performance before and after the seven-day intervention in the IPC and sham groups. Baseline characteristics of the participants are presented in Table 1.

**Table 1:** Baseline characteristics of participants.

Baseline	IPC (*n* = 11)	Sham (*n* = 11)
Height (cm)	166.36 ± 5.57	163.45 ± 3.95
Body mass (kg)	60.72 ± 7.56	57.9 ± 7.16
Systolic blood pressure (mmHg)	104.63 ± 7.03	114.18 ± 20.17
Diastolic blood pressure (mmHg)	71.63 ± 5.07	74.72 ± 10.53

Regarding lower-limb anaerobic performance, seven consecutive days of IPC intervention resulted in significant differences in average power and total work, whereas no significant intervention effects were observed for peak power and fatigue index. Specifically, for average power, the group × time interaction was significant (F(1,20) = 5.45, *p* = 0.03, ${\eta }_{\mathrm{p}}^{2}=0.14$), and the main effect of time was also significant (F(1,20) = 7.66, *p* = 0.01, ${\eta }_{\mathrm{p}}^{2}=0.28$). Post-hoc showed that average power increased significantly after the intervention in the IPC group (pre: 311.14 ± 16.21 W; post: 356.37 ± 15.79 W; *p* = 0.002), whereas no significant pre–post difference was found in the sham group (pre: 338.73 ± 16.21 W; post: 342.57  ± 15.79 W; *p* = 0.76). Similarly, for total work, there was a significant group × time interaction (F(1,20) = 4.99, *p* = 0.037, ${\eta }_{\mathrm{p}}^{2}=0.20$), and a significant main effect of time (F(1,20) = 6.90, *p* = 0.016, ${\eta }_{\mathrm{p}}^{2}=0.26$). Post-hoc indicated that total work increased significantly in the IPC group (pre: 8873.82 ±479.83 J; post: 10,312.64 ± 435.24 J; *p* = 0.003), whereas no significant change was observed in the sham group (pre: 9809.36  ± 479.83 J; post: 9925.46 ± 435.24 J; *p* = 0.78). Conversely, no significant group × time interactions or main effects of group were observed for peak power and fatigue index. However, a significant main effect of time was observed solely for peak power (F(1,20) = 11.23, *p* = 0.003, ${\eta }_{\mathrm{p}}^{2}=0.36$).

### Heart rate and blood lactate responses

**Figure 4:**
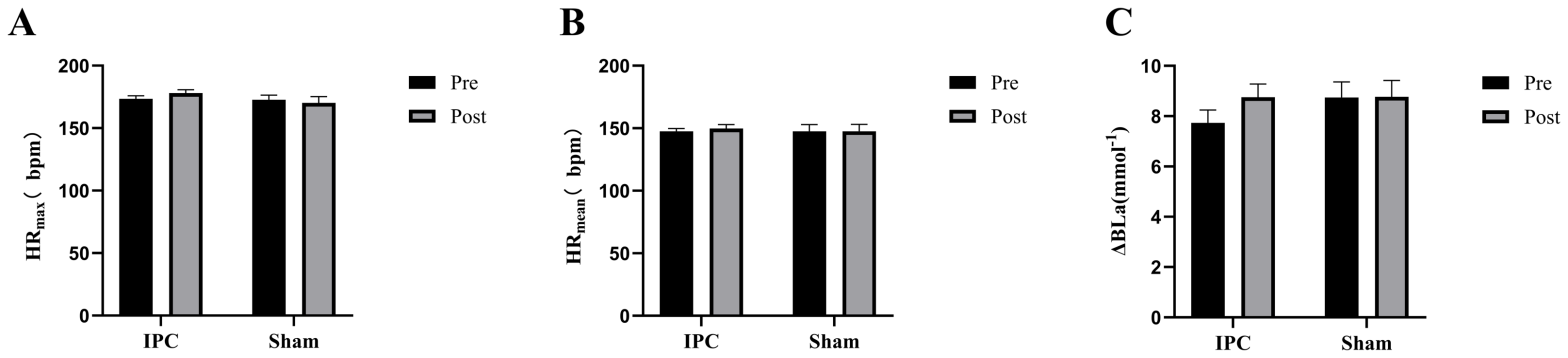
Results of heart rate and blood lactate during the Wingate test before and after IPC. (A) Average heart rate; (B) maximal heart rate; (C) change in blood lactate concentration. HRmean, mean heart rate; HRmax, maximal heart rate; ΔBla, change in blood lactate concentration.

ANOVA revealed no significant effects of the investigated factors on heart rate and blood lactate responses during the Wingate test. Specifically, no significant group × time interactions, main effects of time, or main effects of group were observed for mean heart rate, maximum heart rate, and blood lactate concentration. Detailed data are presented in Fig. 4.

### Lower-limb surface EMG activity

Surface electromyography results indicated no significant differences in the MF of the lower-limb muscles during the Wingate test (Table 2). Specifically, for the vastus medialis, there was a significant main effect of time (F(1,20) = 6.41, *p* = 0.02, ${\eta }_{\mathrm{p}}^{2}=0.24$). Similarly, for the rectus femoris, the main effect of time was significant (F(1,20) = 7.60, *p* = 0.01, ${\eta }_{\mathrm{p}}^{2}=0.28$). No significant main effects of time, main effects of group, or group × time interactions were observed for the remaining muscles, including the vastus lateralis, semitendinosus, biceps femoris, medial gastrocnemius, lateral gastrocnemius, and tibialis anterior.

**Table 2:** Median frequency of lower limb EMG during the Wingate test before and after IPC.

Variable	IPC		SHAM		Main effect		Interaction effect F (p)
	Pre	Post	Pre	Post	Time F(p)	Group F(p)	
VM	51.36 ± 1.19	59.17 ± 2.90	53.01 ± 2.29	55.60 ± 2.09	6.41 (0.02^*^)	0.17 (0.68)	1.62 (0.21)
RF	46.29 ± 4.28	53.28 ± 2.13	50.69 ± 1.38	54.52 ± 1.35	7.6 (0.01^*^)	0.85 (0.37)	0.65 (0.43)
VL	57.86 ± 1.71	56.61 ± 1.66	57.53 ± 1.71	58.43 ± 1.66	0.02 (0.88)	0.13 (0.73)	0.87 (0.36)
BF	57.53 ± 3.33	59.22 ± 1.69	53.94 ± 3.33	55.57 ± 1.69	0.73 (0.4)	1.29 (0.27)	0.0002 (0.99)
ST	66.1 ± 4.17	63.75 ± 3.95	53.45 ± 4.17	57.35 ± 3.95	0.09 (0.77)	3.44 (0.07)	1.47 (0.24)
MG	82.56 ± 4.81	85.47 ± 3.18	77.42 ± 4.81	78.34 ± 3.18	0.5 (0.49)	1.45 (0.24)	0.14 (0.72)
LG	81.65 ± 3.38	81.83 ± 4.32	74.68 ± 3.38	77.39 ± 4.32	0.32 (0.58)	1.37 (0.26)	0.25 (0.62)
TA	69.38 ± 3.47	67.12 ± 2.77	68.31 ± 3.47	74.27 ± 2.77	0.62 (0.44)	0.65 (0.43)	3.06 (0.09)

**Notes.**

* indicates statistical significance at *p* < 0.05.

### Prefrontal cortical oxygenation responses

**Figure 5:**
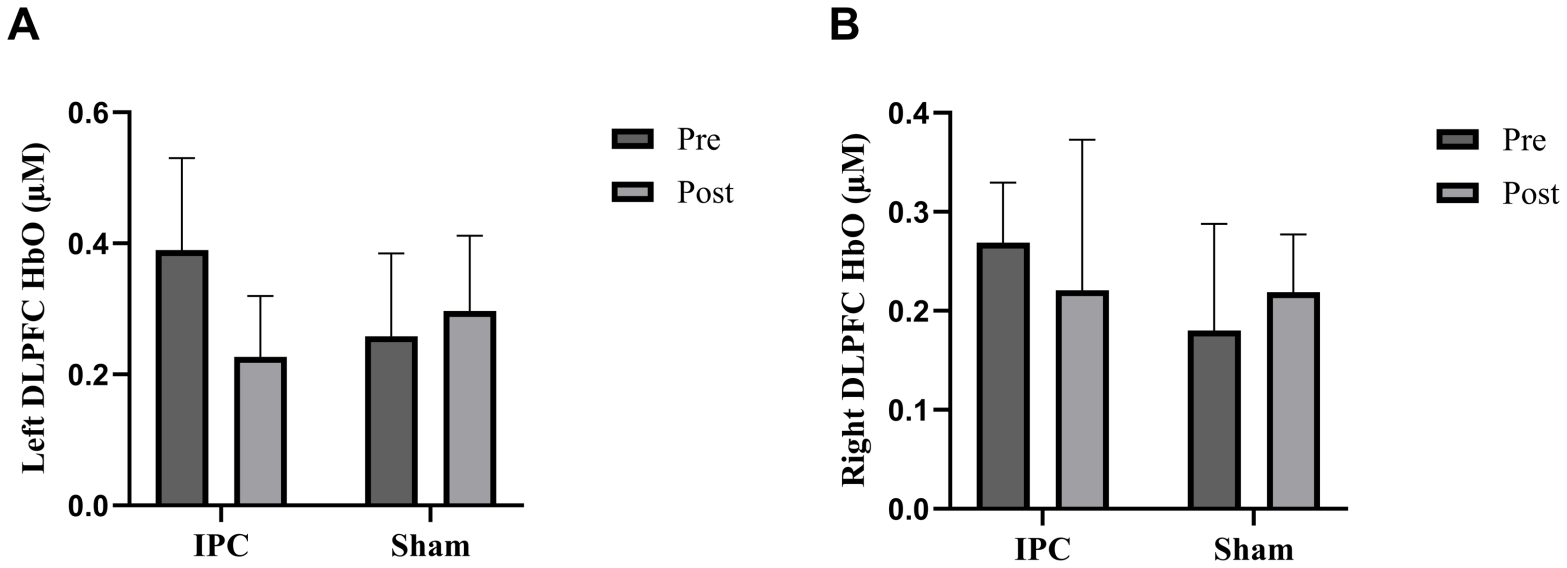
Changes in DLPFC HbO before and after IPC. (A) Left DLPFC; (B) right DLPFC. DLPFC, dorsolateral prefrontal cortex. HbO, oxyhemoglobin.

Regarding prefrontal cortical hemodynamics, there were no significant group × time interactions, main effects of time, or main effects of group for oxyhemoglobin concentrations in either the left or right dorsolateral prefrontal cortex during the Wingate test. Detailed changes in DLPFC HbO are visually presented in Fig. 5.

## Discussion

The primary aim of this study was to determine whether IPC can improve lower-limb anaerobic performance and neuromuscular function in female athletes. The main findings were that a seven-day IPC intervention moderately enhanced lower-limb anaerobic performance, as reflected by increases in average power and total work during a Wingate test. However, contrary to our initial hypothesis, the intervention did not yield group-specific advantages in peak power, nor did it significantly alter prefrontal cortical oxygenation levels or lower-limb neuromuscular activation during the Wingate test.

### Effects of IPC on lower-limb anaerobic performance

Anaerobic capacity reflects the body’s ability to resynthesize ATP *via* anaerobic pathways during brief, intense efforts (Green, 1994). The present results demonstrate that seven consecutive days of IPC enhanced lower-limb anaerobic performance in female athletes, yielding large effect sizes specifically for average power and total work. These ergogenic effects may be related to short-term adaptations induced by repeated IPC stimuli. These findings are partially consistent with those of Patterson et al. (2015), who reported that, compared with placebo, IPC increased peak power during the first three bouts of repeated sprint cycling by 2.4 ± 2.2%, 2.6 ± 2.7%, and 3.7 ± 2.4%, respectively, however, these acute improvements were accompanied by relatively small effect sizes. A recent study also demonstrated that IPC effectively attenuated the decline in repeated anaerobic performance, even under simulated high-altitude hypoxic conditions (Drozd et al., 2025). However, evidence regarding the effects of IPC on anaerobic performance remains mixed. For instance, acute ischemic preconditioning prior to resistance training did not significantly affect upper-limb barbell bench press velocity (Gawel et al., 2025). Similarly, Lalonde & Curnier (2015) found that a single bout of IPC did not significantly alter mean or peak power during a Wingate test in 17 healthy adults. A likely explanation for these discrepant findings lies in the dose of IPC: acute, single-session protocols may not provide sufficient cumulative stimuli to elicit robust improvements in anaerobic capacity; even when statistically significant differences are occasionally observed, they are typically limited to small effect sizes. In contrast, the seven-day IPC protocol used in the present study may have induced more pronounced enhancements in anaerobic work capacity and larger effect sizes. Taken together, these data suggest that repeated IPC sessions may be particularly advantageous for improving anaerobic performance in sports that rely heavily on short-duration, high-intensity energy supply.

### Effects of IPC on heart rate and blood lactate responses

Heart rate and blood lactate responses during the 30-s Wingate test reflect internal load and systemic metabolic stress. In the present study, IPC did not significantly affect the average heart rate (HRavg), maximum heart rate (HRmax), or blood lactate responses. This finding aligns with previous studies indicating that IPC does not alter maximal or mean heart rate during incremental exercise tests (De Groot et al., 2010; ter Beek et al., 2022). During exercise, heart rate increases to meet the elevated oxygen demand of working muscles, and cardiac pump function plays a central role in modulating heart rate responses. Prior work has shown that IPC can improve stroke volume during endurance exercise (Paradis-Deschênes, Joanisse & Billaut, 2018). This effect is thought to be mediated by IPC-induced nitric oxide (NO) release, which promotes peripheral vasodilation, reduces systemic vascular resistance, and improves cardiac pump efficiency. Improved cardiac function may allow the same or greater cardiac output to be achieved at a lower heart rate. In the present study, however, the 30-s Wingate protocol may not have been long enough for heart rate to approach maximal values, potentially masking subtle effects of IPC on cardiac responses. Similarly, IPC did not significantly alter blood lactate accumulation. Blood lactate concentration reflects the balance between lactate production and clearance (Kang & Park, 2016). During short-duration, high-intensity exercise, anaerobic glycolysis dominates energy supply, resulting in rapid lactate accumulation that ultimately contributes to fatigue (Hall et al., 2016). However, recent research indicates that lactate serves as an important circulating carbohydrate fuel providing energy to the body, and blood lactate accumulation *per se* is not the primary factor causing systemic fatigue (Rabinowitz & Enerbäck, 2020). Although no significant group differences in blood lactate were observed here, IPC significantly increased anaerobic power and total work, which suggests that IPC may have potentially contributed to improved performance without increasing metabolic stress.

### Effects of IPC on lower-limb neuromuscular adaptations

Fatigue arises from complex interactions between central mechanisms and peripheral mechanisms at the level of peripheral nerves, neuromuscular junctions, and skeletal muscle (Gandevia, 2001). The MF of the EMG power spectrum is commonly used as an index of muscle fatigue, as fatigue is typically accompanied by a shift of the EMG spectrum toward lower frequencies due to reduced motor unit firing rates and conduction velocity (Fujita et al., 2022). In the present study, IPC did not induce significant improvements in the MF of the lower-limb muscles during the Wingate test. This finding contrasts with previous research by Niespodziński et al. (2021), who reported that after 10 days of IPC, the decline in MF slope during isometric lower-limb contractions was significantly attenuated in 37 healthy men, indicating improved resistance to fatigue. This discrepancy may primarily stem from differences in the load intensity of the exercise tasks. Submaximal isometric contractions involve progressive motor unit recruitment and rely heavily on sustained local oxygen delivery, allowing IPC-induced microvascular benefits to manifest in EMG signals. In contrast, the 30-s Wingate test is a supramaximal all-out effort requiring the immediate recruitment of nearly all available motor units. Such extreme intensity induces rapid and profound peripheral muscle fatigue, likely creating a physiological ceiling effect that masks any subtle IPC-mediated differences in surface EMG spectral shifts.

### Effects of IPC on prefrontal cortical oxygenation

However, IPC did not significantly alter HbO responses in either the left or right DLPFC during the Wingate test. Mechanistically, IPC has been proposed to exert neuroprotective and vasoregulatory effects by modulating NO production, apoptosis-related proteins, brain-derived neurotrophic factor (BDNF), and vascular endothelial growth factor (VEGF) (Guo et al., 2019). To date, however, evidence for IPC-induced changes in cortical activation during exercise remains sparse. One plausible explanation for the absence of significant effects in the present study is the robust capacity for cerebral autoregulation. Cerebral autoregulation refers to the brain’s ability to maintain relatively stable cerebral blood flow (CBF) despite fluctuations in arterial CO_2_ tension, mean arterial pressure, or cerebral perfusion pressure (Shi et al., 2025). This regulation is shaped by multiple overlapping mechanisms—including arterial blood gases (PaCO_2_, PaO_2_), local tissue gas tensions, systemic hemodynamics (blood pressure, posture), cerebral metabolic demand, and neural factors—which act individually and interactively (Zhou, Li & Shen, 2022). A key feature of this system is mechanistic redundancy, whereby multiple pathways can compensate for one another to maintain CBF under high-load conditions (Claassen et al., 2021). Consistent with this notion, Carter et al. (2020) reported that bilateral upper-limb IPC did not significantly affect resting cerebral perfusion, cerebrovascular CO_2_ reactivity, or dynamic cerebral autoregulation. Another possibility is that, under high-intensity exercise conditions, blood flow may be preferentially redistributed away from the brain to support skeletal muscle perfusion. Moreover, high-intensity exercise has been shown to reduce arterial CO_2_ pressure, resulting in cerebral vasoconstriction and decreased CBF (Jia et al., 2024). These mechanisms suggest that the peripheral hemodynamic benefits of IPC—such as increased peripheral blood flow and improved myocardial perfusion (Fan, Shi & Wu, 2023)—may not readily translate into measurable changes in cortical oxygenation during short, maximal efforts.

This study has several limitations. First, the relatively small sample size limits the statistical power, which warrants a cautious interpretation of the findings’ reliability. Additionally, the sample consisted exclusively of female national second-level athletes, which may limit the generalizability of the findings to male athletes, elite professionals, or recreationally active individuals. Future studies should include a broader range of populations to validate the effectiveness of IPC further. Second, the fNIRS measurements were restricted to the DLPFC, which is primarily associated with executive control and inhibitory processes (Bigliassi & Filho, 2022). Motor-related regions, such as the primary motor cortex and supplementary motor area, were not monitored, making it difficult to fully characterise the hemodynamic responses within the sensorimotor network that underlies movement execution, motor unit recruitment, and sensorimotor integration. Future research should consider extending fNIRS coverage to the entire prefrontal cortex and, where possible, motor-related areas. Third, a fixed cuff pressure of 220 mmHg was used for all participants in the IPC protocol. However, the degree of arterial occlusion can be influenced by factors such as cuff material, sex, limb size, and body position. Using a uniform pressure may result in inter-individual variability in occlusion levels and thus in the physiological response to IPC. Future studies should assess individual limb occlusion pressure (LOP) *via* Doppler ultrasound and set cuff pressures relative to each participant’s LOP to enhance the reliability and validity of the intervention.

## Conclusions

In conclusion, a seven-day IPC intervention induced a modest enhancement in sustained lower-limb anaerobic performance in female athletes, specifically by increasing average power and total work during a Wingate test. In contrast, IPC did not significantly affect peak power, median frequency, fatigue index, heart rate, blood lactate responses, or DLPFC HbO levels. Overall, IPC is a safe and effective non-pharmacological intervention that can be incorporated into athletes’ routine training preparation to enhance lower-limb anaerobic performance, particularly in sports requiring repeated high-intensity efforts.

## Supplemental Information

10.7717/peerj.21296/supp-1 Supplemental Information 1 Oxyhemoglobin (HbO) concentration changes in the dorsolateral prefrontal cortex (DLPFC) during the Wingate test

10.7717/peerj.21296/supp-2 Supplemental Information 2 Experimental data Wingate anaerobic performance, surface electromyography (sEMG), heart rate, and blood lactate responses following 7-day RIPC intervention.

10.7717/peerj.21296/supp-3 Supplemental Information 3 Data Codebook for categorical variables and fNIRS file organization

## Additional Information and Declarations

### Data Availability

The following information was supplied regarding data availability: The raw measurements are available in the Supplemental Files.
