# Supplementary material for: Effects of ischemic preconditioning on lower-limb anaerobic performance and neuromuscular adaptations
Source: PeerJ. 2026 Jun 8;14:e21296. doi: 10.7717/peerj.21296 (PMC13256049; doi:10.7717/peerj.21296)
Supplement: Supplemental Information 3 [file peerj-14-21296-s003.docx]

1. Main Dataset (Spreadsheet)：This section describes the variables in the main data file containing physiological and performance metrics.

Variable: Group (Categorical Data)

1 = RIPC Group (Remote Ischemic Preconditioning / Real Stimulation)

2 = Sham Control Group (Sham Stimulation)

2. fNIRS Data Files (Description for the raw CSV files named 01 to 44)

| File Range | Group | Time Point |
| --- | --- | --- |
| 01 – 11 | RIPC Group | Pre-test |
| 12 – 22 | Sham Group | Pre-test |
| 23 – 33 | RIPC Group | Post-test |
| 34 – 44 | Sham Group | Post-test |
